# Nutritional significance of amino acids, vitamins and minerals as nutraceuticals in poultry production and health – a comprehensive review

**DOI:** 10.1080/01652176.2020.1857887

**Published:** 2020-12-13

**Authors:** Mahmoud Alagawany, Shaaban S. Elnesr, Mayada R. Farag, Ruchi Tiwari, Mohd. Iqbal Yatoo, Kumaragurubaran Karthik, Izabela Michalak, Kuldeep Dhama

**Affiliations:** a Faculty of Agriculture, Department of Poultry, Zagazig University, Zagazig, Egypt; b Faculty of Agriculture, Department of Poultry Production, Fayoum University, Fayoum, Egypt; c Faculty of Veterinary Medicine, Forensic Medicine and Toxicology Department, Zagazig University, Zagazig, Egypt; d Department of Veterinary Microbiology and Immunology, College of Veterinary Sciences, Deen Dayal Upadhayay Pashu Chikitsa Vigyan Vishwavidyalay Evum Go-Anusandhan Sansthan (DUVASU), Mathura, India; e Sher-E-Kashmir University of Agricultural Sciences and Technology of Kashmir, Srinagar, India; f Central University Laboratory, Tamil Nadu Veterinary and Animal Sciences University, Chennai, India; g Faculty of Chemistry, Department of Advanced Material Technologies, Wrocław University of Science and Technology, Wrocław, Poland; h Division of Pathology, ICAR-Indian Veterinary Research Institute, Bareilly, India

**Keywords:** Poultry, chicken, nutraceuticals, amino acids, minerals, vitamins, organic mineral, designer food, nanoparticles, health

## Abstract

Nutraceuticals have gained immense importance in poultry science recently considering the nutritional and beneficial health effects of their constituents. Besides providing nutritional requirements to birds, nutraceuticals have beneficial pharmacological effects, for example, they help in establishing normal physiological health status, prevent diseases and thereby improve production performance. Nutraceuticals include amino acids, vitamins, minerals, enzymes, etc. which are important for preventing oxidative stress, regulating the immune response and maintaining normal physiological, biochemical and homeostatic mechanisms. Nutraceuticals help in supplying nutrients in balanced amounts for supporting the optimal growth performance in modern poultry flocks, and as a dietary supplement can reduce the use of antibiotics. The application of antibiotic growth enhancers in poultry leads to the propagation of antibiotic-resistant microbes and drug residues; therefore, they have been restricted in many countries. Thus, there is a demand for natural feed additives that lead to the same growth enhancement without affecting the health. Nutraceuticals substances have an essential role in the development of the animals’ normal physiological functions and in protecting them against infectious diseases. In this review, the uses of amino acids, vitamins and minerals as well as their mode of action in growth promotion and elevation of immune system are discussed.

## 1. Introduction

Nutrition plays a pivotal role in maintaining the health of pullets, quality egg production by laying hens and body growth of broilers (Wang et al. 2017). Nutraceuticals are the nutrients or constituents of animal diet that have nutritional and pharmaceutical importance by preventing various diseases, possessing immunomodulatory potential, providing health benefits and consequently increasing productivity (Dhama et al. 2015; Aronson 2017; Helal et al. 2019; Waheed Janabi et al. 2020). They include nutrients and non-nutrients, like amino acids, minerals, vitamins, fatty acids, enzymes, prebiotics, probiotics, synbiotics, pigments, medicinal herbs, herbal extracts, antioxidants, organic acids, flavouring agents, etc. (Narahari 2014; Alagawany et al. 2018a; Elgeddawy et al. 2020). Nutraceuticals have been in focus in poultry science quite recently due to the nutritional and healthier properties of feed ingredients and the adverse effects of chemical pharmaceuticals like antibiotic resistance and drug residues (Elnesr et al. 2019a, 2020). Amino acids (Ghoreyshi et al. 2019), minerals (Khatun et al. 2019), and vitamins (Ahmad et al. 2019) which are common ingredients of poultry ration or a combination of them (Moghaddam and Emadi 2014; Horvath and Babinsky 2018) can be nutraceuticals, especially important in poultry feeding. Generally, poultry receives nutrients through the consumption of natural feedstuffs, but some key essential amino acids (lysine, methionine, threonine and tryptophan), vitamins and minerals are often offered as synthetic supplements (Ravindran 2010). The refined form of dietary nutraceutical constituents can result in better digestion, absorption, utilization, metabolism and beneficial health effects when compared with conventional forms. Nutraceutical value and conversion efficiency in poultry are influenced by various factors including the bird's genetic potential, environmental conditions, dietary quality and gut health which should be taken into consideration in order to maximize bird productive efficiency (Rinttila and Apajalahti 2013; Sugiharto 2016; Yadav and Jha 2019). For decades, the therapeutic use of antibiotics has been commonly practiced in poultry farms to regulate the ecosystem balance in the gut and to enhance chicken growth (Yadav et al. 2016; Karavolias et al. 2018). This practice has many criticisms such as the increasing prevalence of resistance to antibiotics in birds and the residues remaining in the poultry products (Kabir 2009). Therefore, alternatives to antibiotics have to be found for safe poultry production and promotion of their performances (Dhama et al. 2014a; Yadav et al. 2016; Alagawany et al. 2018b; Abd El-Hack et al. 2020a).

Using nutraceuticals has a beneficial effect, as it gets rid of the adverse effect of antibiotics which led to the elimination of the intestinal microbiota without differentiation between the harmful and useful ones (Sullivan et al. 2001). For example, Rashid et al. (2012) detected Clostridial infection after usage of antibiotics as feed additives. In addition, using antibiotics has several detrimental effects which include the development of antibiotic-resistant genes by intestinal microbiota, propagation of particular intestinal bacteria and digestion changes which can occur due to immune response of internal organs. To preclude these effects, the addition of a high specific substance for a certain infection is needed (Frei et al. 2015). In this condition, nutraceuticals were found to have various beneficial health applications and potential roles in enhancing production performances as they act as antioxidants, safeguard health, modulate gut microbiota, and enhance the immunity of poultry (Rahal et al. 2014, Alagawany et al. 2019; Dhama et al. 2014a; Soomro et al. 2019; Abd El-Hack et al. 2020b). For better delivery, improved bioavailability and utilization of nutraceuticals within the body of poultry, numerous delivery forms are being investigated. Improvised delivery systems of nutraceuticals include chelated, micronized, encapsulated, nano formulated, or chemically modified forms that have prospects for not only better delivery but also conversion efficiency (Aklakur et al. 2016; Helal et al. 2019; Jampilek et al. 2019; Khatun et al. 2019).

This review provides updates with regard to the potential role of amino acids, vitamins and minerals as nutraceuticals that will improve poultry production performance, protect health of birds and enhance immunity, and aid in encountering some public health issues. Also, this review will cover prominent aspects of nutraceuticals used as new strategies to diminish the application of antibiotic growth promoters in poultry diets. Special attention has been paid to the advances in delivery and formulating designer and functional food out of such nutraceuticals.

## 2. Amino acids as nutraceuticals

Amino acids are functional and structural units of protein, nutritionally classified into two groups: non-essential (synthesized in the body) and essential amino acids (cannot be synthesized rapidly enough to meet the metabolic requirement). Amino acids play vital physiological roles in the body (Bortoluzzi et al. 2018; Debnath et al. 2019). After absorption, amino acids are assembled and metabolized to form proteins that are used to build different body tissues. Studies indicated that providing high protein and energy-rich diet to pullets in their growth and egg-laying phase showed positive effects on egg mass and yolk weight (Babiker et al. 2011).

There are differences in recommended essential amino acids levels in various guidelines, which raise concerns for the poultry sector. Extensive research was done on the use of synthetic amino acids in poultry feed. The careful supplementation of synthetic amino acids has the potential to boost the overall amino acid balance and to decrease the level of crude protein in the poultry diet (Waldroup et al. 2005). The use of amino acids in the nutrition lessens the nitrogen loss during metabolism of protein that leads to low excretion of ammonia in the environment and improves growth performance of birds (Kidd and Kerr 1996). Also, in the diet of poultry, amino acids must be balanced to avoid loss of energy that can be diverted to the synthesis of fat (Leeson et al. 1996). Beski et al. (2015) stated that dietary synthetic amino acid supplementation to poultry diets improved feed conversion efficiency and reduced nitrogen excretion. Kidd et al. (2004) indicated that healthy broilers responded positively to the high dietary inclusion of amino acids and had a positive effect on the performance. A study conducted on one day old Cobb male broilers illustrated that dietary supplementation of amino acid chelated trace mineral helped in diminishing levels of circulatory and intestinal heat shock protein 70 (HSP70) and pro-inflammatory cytokine gene expression in heat-stressed broiler chickens. This observation indicates that amino acid chelated trace minerals in the diet can improve gut health by lowering the effect of heat stress (Baxter et al. 2020). There are evidences that amino acid metabolism is affected by health status of birds as shown in some recent studies on challenge vs. non challenge conditions (Chrystal et al. 2020; Hilliar et al. 2020). Diseases like necrotic enteritis affect digestion, absorption and metabolism of amino acids. Low protein diet did not affect predisposition to necrotic enteritis but feeding a standard diet or diet with additional amino acid content can mitigate this disease (Hilliar et al. 2020). Amino acid supplementation favor cecal butyric acid and total short-chain fatty acids production and support growth, development, feed conversion efficiency and improve immunity (Chrystal et al. 2020; Hilliar et al. 2020). Digestibility of amino acids is also affected in health and disease and by alteration of nutrient composition of diet (Keerqin et al. 2017; Chrystal et al. 2020). Diets having 90% arginine compared to lysine and high methionine were found to be beneficial in minimizing oxidative stress, modulating metabolic parameters and influencing indicators of intestinal barrier integrity in turkeys with necrotic enteritis (Ognik et al. 2020).

Ten amino acids classified as essential (lysine, methionine, tryptophan, threonine, arginine, isoleucine, leucine, histidine, phenylalanine and valine) must be provided in the diet for maximum performance. Out of these 10 essential amino acids, lysine and methionine are the first two limiting amino acids for broilers (Corzo et al. 2007; Rehman et al. 2019), while threonine is the third limiting amino acid (Kidd and Kerr 1996). Glycine is considered to be essential for young birds. Glycine and serine are the non-essential limiting amino acids in the diet of poultry (Siegert and Rodehutscord 2019). Cysteine and tyrosine are recognized as semi-essential amino acids because they can be synthesized from methionine and phenylalanine, respectively (Ravindran 2010). The most important amino acids for poultry are listed below.

### 2.1. Methionine

Methionine plays an important role in the optimum growth performance of poultry and is involved in feather synthesis, important biochemical processes (as a methylgroup donator) and muscle accretion (Goulart et al. 2011; Fagundes et al. 2020). This amino acid participates in protein synthesis, methylation reaction of DNA, elimination of reactive oxygen species (ROS) and acts as glutathione (GSH) precursor – a tripeptide, which decreases ROS and thus protects cells from oxidative stress (Kidd 2004; Elnesr et al. 2019b). Therefore, methionine is sensitive to oxidative modification. Dietary methionine inclusion led to better growth performance of broiler chicken at 42 days of age (Wen et al. 2017). The broiler chicks fed with diet containing higher methionine level than National Research Council (NRC) requirements exhibited a significant increase in relative and absolute weight of the breast and significant reduction in abdominal fat (Ahmed and Abbas 2011). The effect of total sulfur amino acid (TSAA) with L-methionine was evaluated on HyLine W36 laying hens’ growth performance, egg production, egg quality including egg weight, egg mass, bone volume, bone mineral content and bone density. The experiment conducted at high temperature, for forty-five weeks revealed that the addition of 85-100% of TSAA in the diet helped birds to alleviate the adverse effects of high temperature (Castro et al. 2019). Thus, the effectiveness of methionine is primarily due to its role as an antioxidant and the effect on the cellular response to the oxidative stress.

### 2.2. Threonine

Threonine is very important for the synthesis and maintenance of proteins in the body and plays a significant role as an essential component of mucin in gut health (Kidd and Kerr 1996; Lien et al. 1997) and is involved in an important metabolic process such as the uric acid formation (Rezaeipour et al. 2012). After absorption of threonine, it is used for gut protein synthesis and protects the gut from anti-nutritional factors and pathogens (Lee et al. 2007). Threonine has a major role in intestinal development and well-functioning (Stoll 2006). This may elucidate the better developed gut in birds fed with higher dietary threonine than recommended levels. Synthesis of mucosal protein and mucin is quicker in the presence of threonine in the lumen which indicates its importance for proper gut functioning (Nichols and Bertolo 2008). Dietary total threonine levels (between 0.70 and 0.93%) led to the optimum gut morphology (Zaefarian et al. 2008). Significant improvement was detected in performance indices [body weight (BW), body weight gain (BWG), dressing percentage, relative breast weight)] of birds fed with a diet supplemented with threonine compared with those fed with a diet without threonine supplementation (Al-Hayani 2017). Valizade et al. (2016) stated that a higher level of threonine at 0.843% could be the required level that may result in optimal growth performance. Dozier et al. (2001) suggested that the positive effects of threonine supplementation on the performance of broilers may be due to the participation of threonine in the development of intestinal mucosa and also in the function of the digestive enzymes. Supplementation of threonine improves carcass characteristics probably because threonine is the second limiting amino acid for breast meat yield (Estalkhzir et al. 2013). The applications of threonine, above NRC requirements, resulted in a better growth rate, feed utilization, carcass quality and gut health and increased ileal digestibility of amino acids and protein, and enhanced immunity (Ahmad et al. 2020). In broilers, Zarrin-Kavyani et al. (2018) found an improvement in feed intake through the grower period and an improvement in BW throughout the grower and overall period, whereas a better feed conversion ratio (FCR) through the starter period in birds fed with 10% extra threonine in comparison with the control diet. Thus, methionine has a major role in improving the intestinal health of the birds thereby increasing the performance of the birds.

**Figure 1.**
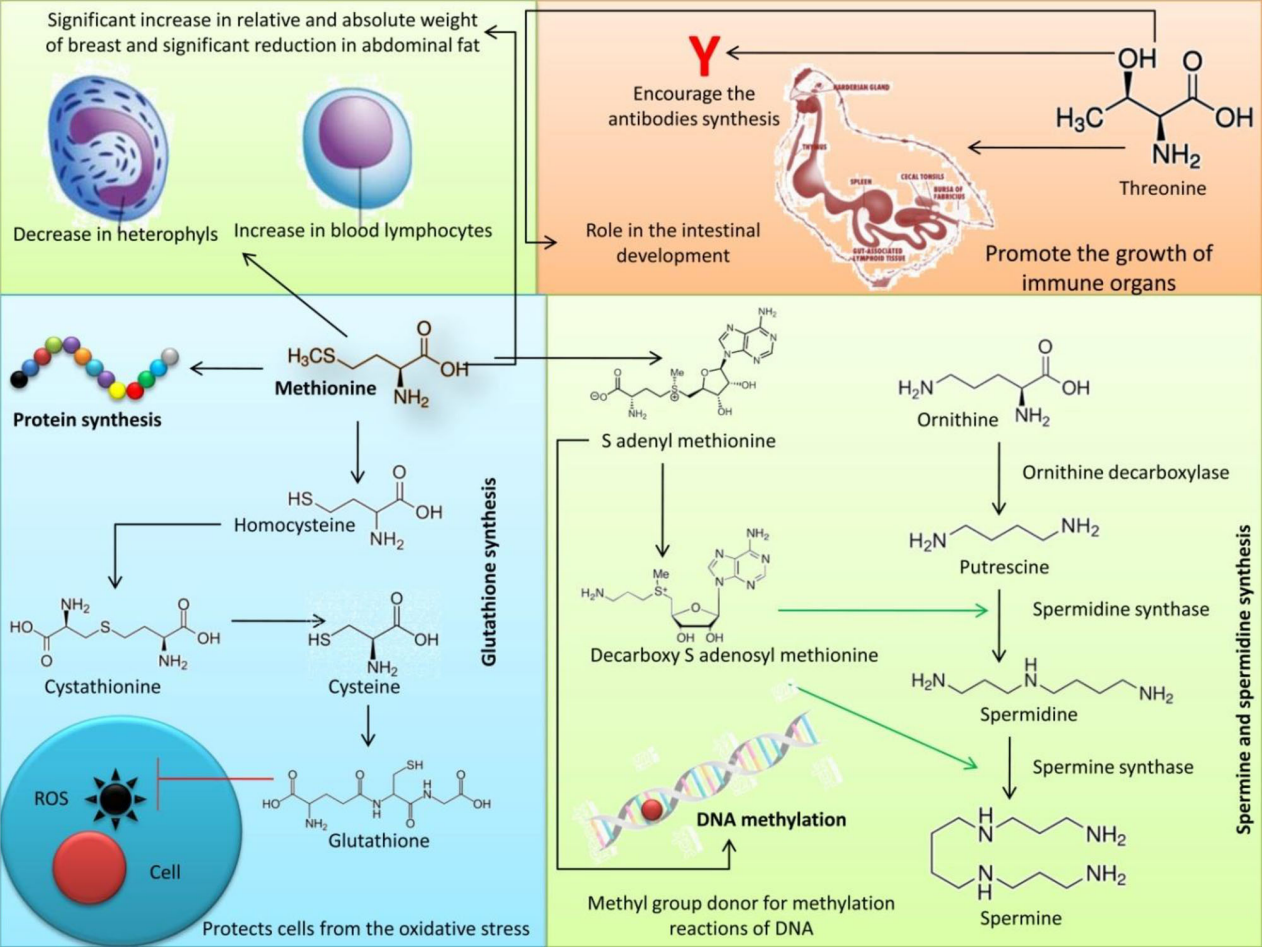
Effect of aminoacids – methionine and threonine on poultry health: (1) Methionine participates in synthesis of protein, (2) Methionine is a glutathione precursor, (3) Methionine is required for the polyamines (spermine and spermidine) synthesis that takes part in nucleus and cell division events, and (4) Methionine is the most important methyl group donor for methylation reactions of DNA and other molecules.

An overview of the effects and modes of actions of the amino acids methionine and threonine on poultry health is depicted in Figure 1.

### 2.3. Cysteine

Cysteine serves as a semi-essential amino acid because it can be synthesized from methionine and serine by trans-sulfuration (Stipanuk 2004). Therefore, the requirement for this amino acid is usually considered together with methionine and cysteine (Goulart et al. 2011). It plays critical roles in protein structure and function and in protecting against oxidative stress. This amino acid acts as a precursor of some constituents that play a central role in the antioxidant protection system of the body such as glutathione (Mari et al. 2009; Jong et al. 2012). Glutathione is a related metabolite of cysteine directly, and of methionine indirectly. In addition to methionine, cysteine can improve intestinal histomorphometric indices of broiler chickens (Elwan et al. 2019), leading to the increase in the absorption of nutrients. Thus cysteine can prevent from oxidative damage.

### 2.4. Arginine

Arginine must be sufficiently available in the chicken diet to maintain the immunological and physiological functions and to support protein accretion (Khajali and Wideman 2010). This amino acid can be used as a potential immunomodulating agent to improve the immune function of broiler chickens (Xu et al. 2018). The dietary arginine level [120% according to NRC (1994) recommendation] should support the proper functions of immune system in healthy chicks (Kidd et al. 2001). Arginine increases the specific immune response against Infectious Bursal Disease (IBD) in chickens (Tayade et al. 2006). The addition of arginine in the broiler diet could improve the growth performance of chickens at 42 days of age (Xu et al. 2018). The supplementation of arginine in the diet reduced the percentage of abdominal fat by suppressing the activities of glucose-6-phosphate dehydrogenase, fatty acid synthase (lipogenic enzymes) and malate dehydrogenase in the liver of meat-type ducks (Wu et al. 2011). It can be concluded that arginine improves the immunity in poultry.

### 2.5. Glutamine

Glutamine is known to provide nitrogen and energy source for the proliferation of immune and intestinal mucosal cells and is required together with cysteine to synthesize antioxidants such as glutathione (Newsholme 2001; Obled 2003). Glutamine is also an essential nutrient for animals under stress conditions, such as infection, injury, high temperature (Soltan 2008). This amino acid plays an important role in the case of necrotic enteritis disease that causes significant economic loss in the broiler chicken industry. L-glutamine compensates for metabolic losses from this infection, improves the intestinal development and gut morphology, improves growth performance and serum biochemical indices (Soltan 2008; Xue et al. 2018). Collectively, glutamine is a non-essential amino acid, but in disease conditions, it works as an essential amino acid and plays a significant role in the improvement of immunity and metabolism in the body.

### 2.6. Lysine

Lysine is one of the limiting amino acids in the poultry diet. Nasr and Kheiri (2011) illustrated that additional lysine at the level of 120% of NRC in Arian broiler diets optimized BWG, whereas a low level of lysine decreased growth and live weight. There are positive effects on meat yield and growth performance in response to supplemental lysine and methionine in male broiler diets from 21 to 41 days of age (Zhai et al. 2016). To differ with this, another study showed that the dietary supplementation of digestible lysine and threonine did not influence the performance and egg quality of laying hens markedly as no visible changes in egg production, total solids in albumen and yolk, specific gravity or percentage of albumen and yolk were noticed (Figueiredo et al. 2012). Lysine was found to improve carcass quality and growth performance of broilers (Belloir et al. 2019). Finally, the main role of lysine is to participate in protein synthesis and cell growth and maintenance, and is considered as a reference amino acid in the ideal protein diet.

### 2.7. The role of amino acids in the immune system

The health status of birds is directly related to their immune system, whereas birds with an adequate immune system grow better. The majority of essential amino acids are perceived as critical resources for cytokine production and immune function (Kidd 2004; Li et al. 2007). Thus, the demand for essential amino acids is likely to increase in the presence of immune stress or inflammation (Le Floc'h et al. 2004). Also, amino acids are associated with the antibody production in animals (Han and Lee 2000). Adequate provision of dietary amino acids is required to maintain normal immunocompetence and protection of the host from some diseases in all species (Beski et al. 2015). Therefore, the development of immune function in poultry will be promoted if they receive sufficient amino acids in their diets. Research has shown that birds display better performance at higher dietary threonine and methionine levels with an improved immune system (Yaqoob and Ali 2018). Threonine works as a proteinogenic amino acid and also is part of immunoglobulins. Increased threonine concentration in the diet may promote the growth of immune organs, encourage the antibody synthesis and mitigate the immune stress caused by Newcastle disease (ND) virus or *Escherichia coli* challenge (Azzam and El-Gogary 2015; Trevisi et al. 2015). Also, Mandal et al. (2006) clarified that broilers fed with a diet containing 1.02% of threonine had 17% greater bursa weight, 7% greater thymus weight and 16% greater spleen weight when compared to those fed with the control diet containing 0.96% of threonine. Tryptophan used as a supplement, due to its requirement for protein synthesis, also works as a precursor of serotonin, a neurotransmitter involved in the regulation of feed intake (Kerr et al. 2005).

The regular diet may not accommodate the growing bird’s requirements; therefore early feeding with amino acids is necessary as their oxidation rate increases during the inflammation (Jha et al. 2019). An inadequate level of threonine in the diet may affect the production of immunoglobulins in broiler chickens because threonine is their integral part (Azzam and El-Gogary 2015). Supplemental lysine or methionine can stimulate the immune responses of broilers (Faluyi et al. 2015; Saleh et al. 2018). The levels of lysine and methionine treatments (30 and 40% more than NRC recommendation, respectively) led to a significant decrease in heterophils and an increase in blood lymphocytes and heterophils: lymphocytes ratio as a stress index (Bouyeh 2012). Bouyeh (2012) indicated that methionine plays four main roles related directly or indirectly to immune system responses: (1) participation in synthesis of protein, (2) as a glutathione precursor, (3) required for the polyamines (spermine and spermidine) synthesis that take part in the nucleus and cell division events, and (4) is the most important methyl group donor for methylation reactions of DNA and other molecules. The dietary addition of methionine, cysteine and arginine boosted BWG and plasma IGF-I levels in young chicks (Kita et al. 2002).

Thus, amino acids are beneficial in improving the general physiological status (Bouyeh and Gevorgyan 2016), immunizing against infectious diseases (Faluyi et al. 2015), and stabilizing under noninfectious or managemental conditions (Saleh et al. 2018) thereby enhancing the production performance of birds (Ghoreyshi et al. 2019). Previous studies showed that broilers challenged with any infection had poor performance because of structural and functional changes that occured in the intestinal mucosa during the infection (Su et al. 2015; Gottardo et al. 2016). Enteric infections in broilers may have a large influence on the endogenous amino acids losses within the gastrointestinal tract (GIT). Gottardo et al. (2017) stated that the diet supplementation with amino acids (glutamine, arginine and threonine) above the recommended levels for growth may be necessary to improve the immune response against *Eimeria* and *E. coli*. Tan et al. (2014) found a significant increase in density and number of goblet cells in the jejunum of birds challenged with a coccidiosis vaccine supplemented with arginine. Dietary supplementation with free methionine mitigates intestinal oxidative stress induced by *Eimeria* spp*.,*in broiler chickens (Khatlab et al. 2019). Methionine plus cysteine are additionally required to support immune response to pathogenic *Eimeria* spp*.,* and therefore additional supplementation helps confer resistance to *Eimeria*-infected chickens (Maroufyan et al. 2013). Thus, amino acid metabolism is different depending on health status in birds (challenge vs. non-challenge conditions) (Chrystal et al. 2020; Hilliar et al. 2020). Digestibility, absorption and metabolism of amino acids is affected in health and disease. Their deficiency can predispose and their supplementation can prevent or mitigate disease (Hilliar et al. 2020). Suppplementation of amino acids has resulted in production of cecal butyric acid and total short-chain fatty acids production and support growth, development, feed conversion efficiency and improve immunity (Chrystal et al. 2020; Hilliar et al. 2020). Nutrient composition of feed also affects metabolism of amino acids (Keerqin et al. 2017; Chrystal et al. 2020).

## 3. Vitamins as nutraceuticals

Researchers have made important advances in understanding the significance of vitamin adequacy to sound poultry nutrition. Vitamins are essential nutraceuticals, required for the optimum general health and physiological functions such as development, growth, maintenance and reproduction. Vitamins exert catalytic functions that facilitate nutrient synthesis, thus controlling metabolism and affecting the performance and health of poultry. Vitamins in poultry feeds have two origins; they are natural components of the ingredients used to prepare the diet and they can be added as a supplement in a concentrated form (Whitehead 2002). There are many vitamins (fat-soluble vitamins: A, D, E and K; and water-soluble vitamins: B_1_, B_2_, B_6_, B_12_, folic acid, pantothenic acid, biotin, niacin and vitamin C) needed for optimal poultry health. The use of these nutrients in sufficient quantities can improve animal health. Most vitamins cannot be synthesized by birds and must be provided by feed, however, the feed alone is not sufficient to cover vitamin requirements. Diets supplemented with vitamins play an important role in disease treatment and prevention; because vitamins allow an animal to use proteins and energy for health improvement, FCR, growth, and reproduction (Whitehead 2002; McDowell and Ward 2008).

If vitamins are absent from the diet or improperly absorbed or utilized, specific diseases or deficiency syndromes occur. Deficiency of vitamins might cause disease states in poultry. Ruffled plumage, cessation of growth, incoordination, weakness, ataxia, xerophthalmia and blindness occur due to deficiency of vitamin A. Exudative diathesis and encephalomalacia are seen due to deficiency of vitamin E. Polyneuritis, perosis, impairment of food utilization and curled toe paralysis occur due to deficiency of vitamin B complex, and anaemia due to folic acid and vitamin B_12_ deficiency. There are some vitamins such as vitamin B_12_, folic acid, pantothenic acid and biotin, etc. which are essential for the normal development of the hemopoietic organs and erythropoiesis. Ferdous et al. (2018) stated that vitamins may be used with drinking water to get good results in BWG, hematological indices and biochemical profiles without any harmful effects on broiler chickens. Vitamins may improve the development of the intestinal mucosa and protect enterocytes from proapoptotic oxidant stress (Hassanpour et al. 2016). The proper ratios of the fat-soluble vitamins and the combination of the four vitamins – A, D, E and C, as a vitamin emulsion positively affected the performance of broiler chicks (Kamalzadeh et al. 2009). In summary, vitamins improve the physiological and health status of birds.

### 3.1. Vitamin E

Vitamin E (α-tocopherol) is a biological antioxidant and contributes to the improvement of growth performance and physiological and immunological status of broiler chickens due to its ability to reduce lipid peroxidation and neutralize free radicals in both skeletal muscle and plasma (Gao et al. 2010; Selim et al. 2013). Vitamin E, selenium and carotenoids are the prime antioxidant components in poultry feed (Surai and Kochish 2019). Weber (2009) reported that deficiency signs of clinical vitamin E include exudative diathesis, muscular myopathy and encephalomalacia in chicks (disturbance of the nervous system), as well as some subclinical vitamin E deficiency such as slow growth performance, diminished fertility and frequent health problems. Therefore, the antioxidant properties of vitamin E were investigated regarding its vital role in the prevention of diseases that occur due to lipid peroxidation and protein oxidation via a free radical mechanism (Colombo 2010; Rizvi et al. 2014). Moreover, vitamin E has a significant role in the improvement of health by boosting both humoral and cell-mediated immune functions (Rizvi et al. 2014). Vitamin E protects the phospholipids of sub-cellular and cellular membranes from the destruction by the lipid oxidation and therefore maintains the functionality and morphological integrity of tissues and cells of the organism (Weber 2009). Vitamin E has been found to improve antioxidant defense, immune response and physiological functions of birds (Habibian et al. 2014; Min et al. 2018). It may have an effect on gene regulation e.g., glutathione peroxidase (GSH-Px) gene (Min et al. 2018).

Dietary vitamin E supplementation for commercial broilers significantly improved the immune response and antioxidant concentrations in the liver (Karadas et al. 2016). Also, α-tocopherol helps in the resistance and prevention of many diseases through its modulatory effect on the immune system by the macrophage activation and antibody production (Weber 2009). The levels of dietary vitamin E (40 or 80 IU/kg feed) may alter the immune function, including the innate cellular oxidative immunity of broiler chickens (Perez-Carbajal et al. 2010). The direct impact of vitamin E on the immunological system is through inhibiting protein kinase C in the lymphocyte and monocyte cells, and decreasing the secretion of immunosuppressive factors such as hydrogen peroxide (Erf et al. 1998). Also, the improved immune response by supplementation of vitamin E in broiler chickens may be due to its antioxidant properties and ability to reduce concentrations of plasma corticosterone (Puthpongsiriporn et al. 2001). Vitamin E-rich diet may reduce stress by suppressing the catabolic response in the body, causing improvement of the production indices, including increased BW (Rymer and Givens 2005). Choct and Naylor (2004) revealed that the use of vitamin E in the diet decreased the mortality rate of male broiler chickens. As an important micronutrient, vitamin E optimizes the reproduction and performance of farm animals. Moreover, it safeguards the ovarian follicles from oxidative damage and also has an important function in egg production by facilitating the yolk precursor (vitellogenin) release from the liver (Weber 2009).

Vitamin E (2 g α-tocopherol acetate/kg feed) augmented carcass mass and decreased the content of the abdominal fat of broilers (Zaboli et al. 2013). This form of vitamin E constitutes the second line of antioxidant defense in biological systems, and is the main lipid-soluble antioxidant, breaks the chain of lipid peroxidation in the membrane of cells and prevents the lipid hydroperoxides formation (Halliwell 1987). There is a favorable influence of vitamin E on the sensory and technological quality of meat (Ryu and Kim 2005). Zdanowska-Sasiadek et al. (2016) illustrated that vitamin E addition in the diet had a significant influence on chicken meat quality by reducing juice drip and increasing the water-holding capacity of meat. Improved meat quality is reflected in higher sensory grades. Finally, vitamin E plays a role in growth, immunity and the protection of biological systems against oxidative damage as well as in meat and meat products. Thus vitamin E acts as antioxidant, improves immunity, fertility, growth and development in poultry.

### 3.2. Vitamin D

Vitamin D_3_ is created naturally by the sunlight action on the skin of most mammals and all birds. Vitamin D_3_ is an important nutrient for bone growth and has a critical role in biological pathways such as immune function, calcium (Ca) homeostasis and cellular proliferation and differentiation (Holick 2004). Also, vitamin D is associated with various physiological processes, including bone mobilization and mineralization and phosphorus (P) and calcium absorption (Garcia et al. 2013). Supplementation of vitamin D induces the intestinal absorption of phosphorus and calcium, encouraging the production of calcium-binding protein in the mucosa, activating the calcium-activated tenderisation complex by the increase in the plasma calcium concentration (Garcia et al. 2013). Also, it increases the re-absorption of Ca and P in the renal tubules and impacts the calcification process by boosting the uptake of minerals by bones (Weber 2009). Higher levels of vitamin D in the diet increase absorption of Ca and P and improve bone strength and consequently leg health (Browning et al. 2012). Additionally, vitamin D regulates the parathyroid hormone secretion and stimulates many tissues with vitamin D receptors. Therefore, deficiency of this vitamin can lead to decrease in productivity and the appearance of metabolic disorders (Garcia et al. 2013). The dietary addition of 25-hydroxyvitamin D (25(OH)D_3_) decreased the incidence of tibial dyschondroplasia (Fritts and Waldroup 2003) and had an affirmative influence on the quality of bone in broiler chicks (Świątkiewicz et al. 2006). Also, Driver et al. (2006) stated that the vitamin D_3_ addition alleviated the clinical signs of tibial dyschondroplasia disease by inducing maturation of chondrocytes. In laying hens, vitamin D plays a role in the optimal function of the skeletal system, strengthening the claws, beak and bones. It also has a positive impact on the quality of eggshells produced by layers. Shojadoost et al. (2015) revealed that 1,25-dihydroxyvitamin D_3_ (1,25(OH)_2_D_3_) has an immunomodulatory property in chicken macrophages. Rodriguez-Lecompte et al. (2016) indicated that vitamin D induced upregulation in the expression of both pro- and anti-inflammatory cytokines. Therefore, the presence of high doses of vitamin D_3_ or its derivative 25(OH)D_3_ above the recommended levels has a positive influence on the immune system particularly when dietary levels of calcium are low. Irrespective of form, the apparent total tract digestibility of calcium was higher in diets enriched with vitamin D. The apparent total tract digestibility of phosphorus was higher in 3,000 IU/kg feed of vitamin D_2_ compared to the other treatments. The utilization of calcium and phosphorus by laying birds can be enhanced by the addition of different sources of vitamin D in rations (Adhikari et al. 2020). Finally, the deficiency consequences of this vitamin are serious, including rickets, poor growth and immune response and also reduction of the production. Thus vitamin D can support bone growth and development, immunity and stabilize calcium-phosphorus metabolism in poultry.

### 3.3. Vitamin K

Vitamin K regulates the production of some coagulation factors in the blood such as prothrombin and clotting factors (VII, IX and X) which are involved in stopping uncontrolled bleeding from wounds. Therefore, deficiency of this vitamin increases blood-clotting time leading to hemorrhagic diseases in organs and tissues. Also, vitamin K is important in relation to bone formation and re-modeling which may be due to the fact that osteocalcin (one of the main bone proteins) depends on vitamin K (Weber 2009). Vitamin K-dependent carboxylation of bone matrix proteins is regarded as important for bone matrix calcification (Gundberg and Nishimoto 2006). Fleming et al. (2003) established that additional vitamin K_3_ (10 mg/kg feed) in the diet led to higher proximal tarsometatarsus cancellous bone volumes of laying hens.

Zhang et al. (2003) conducted a study on male broiler birds for seven weeks to assess the effect of dietary vitamin K levels on bone quality and growth performance. The result of this experiment advocated the inclusion of 8 mg/kg feed and 2 mg/kg feed of vitamin K in the diet of starter and grower broilers, respectively. Vitamin K supplied in different concentrations improved the carboxylation of osteocalcin and increased the hydroxyapatite binding ability of serum osteocalcin and therefore improved the bone quality (Zhang et al. 2003).

In contrast to this, some researchers have investigated the effect of vitamin K deficient diet supplied to the laying hens for a time period of 28 weeks. Reduction in the concentration of skeletal/bone protein gamma-carboxyglutamic acid (Gla) and altered blood clotting was observed. But despite of the insufficient vitamin K level, no significant adverse effects on skeletal metabolism in laying hens, their growing progeny embryos and young chickens were noticed (Lavelle et al. 1994). The effect of vitamin K supplementation in hen's diet on hatchability was also studied (Panda and Pradhan 1967). Thus, vitamin K improves bone development, growth performance, blood clotting and egg development in poultry.

### 3.4. Vitamin A

Vitamin A is necessary for the visual development, growth, reproductive physiology, and maintenance of the integrity of epithelia and the skeleton (Weber 2009). Also, it supports an optimum immune response and thus diminishes the susceptibility to infection. Supplementation of vitamin A at a level higher than recommended by NRC (1994) is preferable to aid normal development of the reproductive organs and membrane integrity of laying hens under heat stress (Kaya and Yildirim 2011). Abd El-Hack et al. (2017a) emphasized the effectiveness of vitamin A at the level of 16,000 IU/kg diet in improving the productive performance parameters. Vitamin A addition to the diet can prevent inhibition of growth performance in poultry that may be deficient in this vitamin (Yuan et al. 2014). Vitamin A levels required to maximize immunocompetence have been displayed to be much higher than that necessary for the feed efficiency and optimum growth (Friedman and Sklan 1997). Dietary vitamin A at a high level of 12,000 IU/kg feed augmented the antibody titer against Newcastle disease virus of hens under heat stress (Lin et al. 2002). Vitamin A is necessary for the epithelial tissue integrity that represents the main defense against the entry of pathogens. Also, Vitamin A is useful in increasing antibody synthesis against pathogens that are able to get into the body (Das et al. 2011). Vitamin A encourages antibody responses to T-cell–dependent antigens (Ross 2012) and induces protective antitumor immunity by some mechanisms such as enhancement of migration to lymph nodes and induction of cell differentiation (Mullin 2011). Additionally, vitamin A under heat stress is a vital antioxidant that minimizes lipid peroxidation (Abd El-Hack et al. 2015). Its supplementation in female quail’s diet improved the development and growth of the reproductive system accompanied by high levels of follicle-stimulating hormone (Fu et al. 2000). Generally, vitamin A improves the productive performance, immunity and reproductive system of poultry.

### 3.5. Vitamin C

Vitamin C (ascorbic acid) increases disease resistance in birds by strengthening the immune system. It plays a significant role in the biosynthesis of corticosterone, a hormone that enhances energy supply during stress (Ahmadu et al. 2016). Of note, poultry can produce vitamin C (Maurice et al. 2002). Ascorbic acid is synthesized in the kidney in birds, and in the liver in some mammals (Ahmadu et al. 2016). The endogenous production of this vitamin is usually considered as not sufficient for the biological demands in poultry, especially during severe environmental conditions (Pardue and Thaxton 1986). Therefore, classical deficiency of this vitamin does not take place in poultry, but it has been shown that additional ascorbic acid has positive effects under stressful conditions.

Sahin et al. (2001) stated that vitamin C increased performance and could improve carcass traits in birds reared under heat stress. Dietary vitamin C supplementation (200 mg/kg feed) provided protection against the risk of high stocking density and improved final BW, reduced mortality percentage and down-regulated HSP70 expression level in the liver (Shewita et al. 2019). Vitamin C (100 and 200 mg/kg feed) exerted a positive influence on laying, egg fertilization and hatchability indices (Nowaczewski and Kontecka 2005). Vitamin C improves the absorption of iron (Fe) leading to increase in the hemoglobin level and red blood cells (Moura and Pedroso 2003). The supplementation of 200 mg/kg feed of ascorbic acid was beneficial for improving immunity and performance and for exploiting the full genetic potential of the commercial broilers (Lohakare et al. 2005). Also, vitamin C plays a major role in cellular antioxidant defenses (Ahmadu et al. 2016). Seven (2008) stated that this vitamin acts as an antioxidant by reacting with all oxygen species and the formation of dehydroascorbyl (a particular inert radical), as well as by transferring radical equivalents from lipid phases. Through regulation of gene expression like GSH-Px gene, vitamin C has been found to prevent oxidative stress, improve immune response, and modulate physiological functions (El-Senousey et al. 2018; Min et al. 2018). Finally, the effectiveness of vitamin C is primarily due to its potent role as an antioxidant. Therefore, it is very important in poultry farms in high-temperature zones due to its important role in alleviating stress.

### 3.6. B Vitamins

B vitamins have very important functions in metabolism of poultry, as most of them represent coenzymes that fuse with larger enzyme molecules to accelerate many metabolic processes. Vitamins B_1_, B_2_, B_6_, biotin, pantothenic acid and niacin are involved in energy metabolism, but folic acid and vitamin B_12_ exert their activity in the cell and growth maintenance (Weber 2009).

#### 3.6.1. Thiamin

Thiamin (vitamin B_1_) is actively and rapidly absorbed from the small intestine and then is transformed by phosphorylation into the active coenzyme – thiamin pyrophosphate that is involved in the oxidative decarboxylation of ketoglutaric acid and pyruvic acid (Chen et al. 2018a). The reactions generate succinyl-CoA and acetyl-coenzyme A (CoA) that are involved in proteins, lipids and carbohydrates metabolism (Haas 1988; Hamano et al. 1999). Weber (2009) summarized some deficiency symptoms of thiamin in poultry that included weight and appetite loss, weakness, heart failure (sudden death syndrome), fatty degeneration of the liver, mucosal inflammation, atrophied ovaries and reduced egg production.

#### 3.6.2. Vitamin B_6_

Vitamin B_6_ (pyridoxine) plays an important role in the metabolism of fatty acids, carbohydrates and amino acids and displays a critical function in the production of energy by the citric acid cycle (McDowell 1989). Pyridoxine is functionally important as pyrodoxal phosphate (co-factor of various enzymes) in the transformation of amino acids and assists in the synthesis of proteins required for immune responses (Hossain et al. 1998). Some studies have reported the importance of vitamins during embryonic development. *In ovo* vitamin B_6_ administration (40, 60, 80 and 120 µg/egg) significantly augmented the hatchability percentage in Japanese quail (Elsayed et al. 2010). Also, *in ovo* injection of vitamin B_6_ (100 µg/egg) significantly increased BW at 28 days of age (Bhanja et al. 2012). Vitamin B_6_ is involved in the erythrocytes formation and the activities of growth hormone, insulin, thyroid, gonadotropic and adrenal hormones (El-Kholy et al. 2019). Vitamin B_6_ is essential for brain development and function and benefits the body to synthesize serotonin, melatonin and norepinephrine hormones (Pond et al. 1995).

#### 3.6.3. Riboflavin

Riboflavin is an essential constituent of two major coenzymes, flavin adenine dinucleotide (FAD) and flavin mononucleotide (riboflavin-5′-phosphate). The coenzymes play major roles in the development, growth, cellular function and energy production and metabolism of steroids, fats, and drugs (Rivlin 2010; Said and Ross 2014). This vitamin is phosphorylated in the mucosa of the intestine to flavin mononucleotide during absorption and then converted in the liver to FAD. Riboflavin is an essential factor of flavin enzymes (flavoproteins) that are involved in the transfer and transport of hydrogen inside the respiration chain and consequently contributes to energy production (Weber 2009). Riboflavin supports the maintenance of the normal concentration of homocysteine in the blood (Rivlin 2010). It is required for the proper functioning of the cellular antioxidant protection, metabolism, and nervous system in chickens (Belinda 2014). As such, riboflavin is a vitamin that is required for the growth and overall good health in poultry.

#### 3.6.4. Vitamin B_12_

Vitamin B_12_ belongs to a specific group of cobalt containing coronoids with biological activity in animals and humans. It is available commercially for addition to the feed as cyanocobalamin. It is an essential constituent of some enzyme systems that carry out a number of basic metabolic functions in the body (McDowell 1989). This vitamin plays a central role in the homocysteine metabolism, energy metabolism, blood function and the immune system. Ahmad et al. (2019) stated that vitamin B_12_ works as a co-factor for methionine synthase and L-methylmalonyl-CoA mutase, and improved ducks hematological parameters such as white and red blood cells and their well-being. Vitamin B_12_ plays a central role in the normal functioning of the nervous system and brain as well as regulation and creation of nucleic acids (DNA and RNA) (Ahmad et al. 2019). Moreover, it participates in fatty acid metabolism and energy generation. Erythrocytes require this vitamin for their proliferation and maturation, therefore, erythrocytes lacking vitamin B_12_ cannot be mature what can lead to hemolysis and hyperbilirubinemia (Zittoun and Zittoun 1999; Khanduri and Sharma 2007), which may cause cardiovascular diseases and depress immunity.

**Figure 2.**
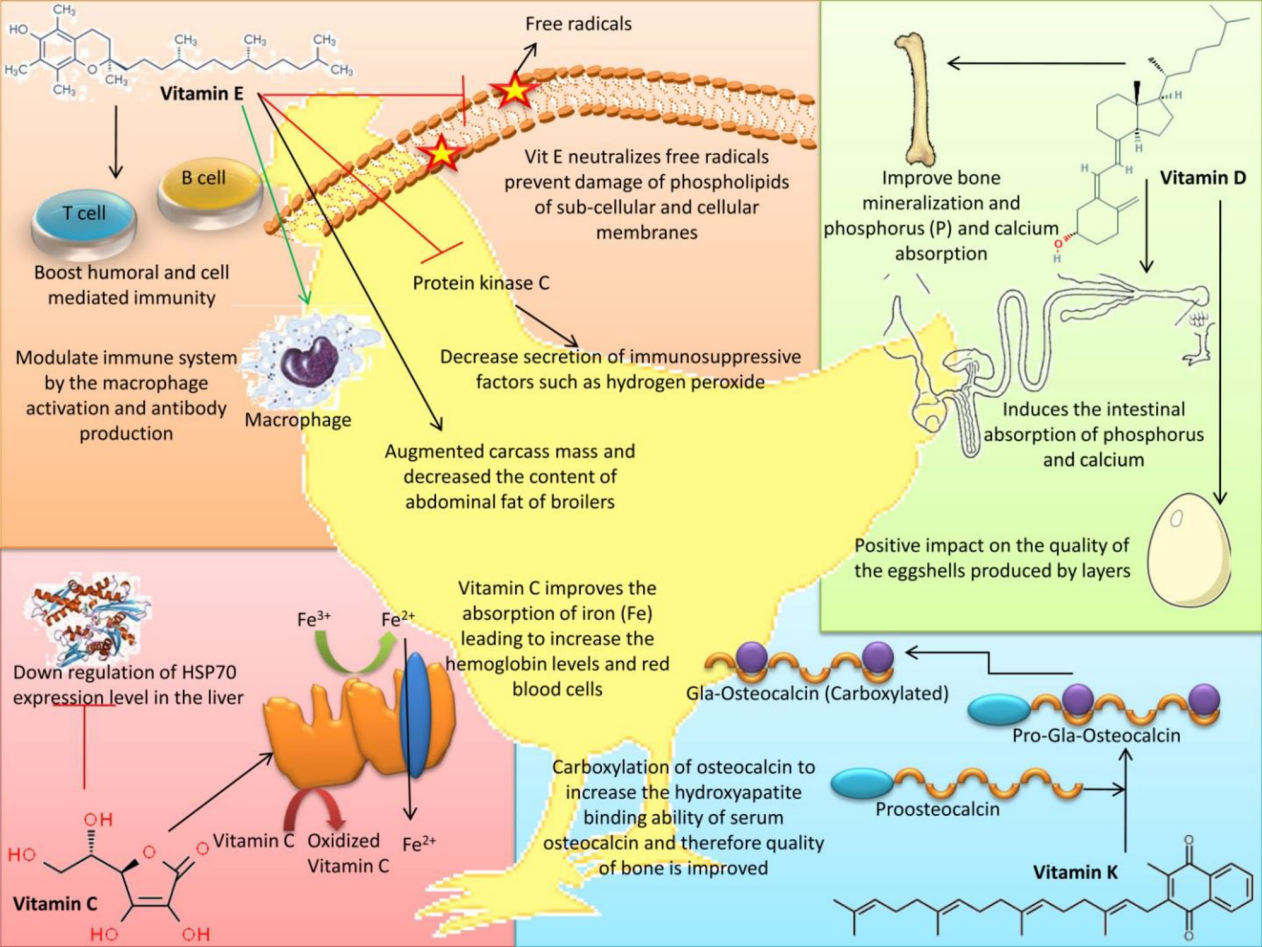
Beneficial effects of different vitamins on poultry health.

An overview on the modes of action with regard to the beneficial effects of different vitamins on poultry health is depicted in Figure 2.

## 4. Minerals as nutraceuticals

The performance and health of birds are the main factors affecting the profits of poultry producers. The recent state-of-the-art opinion points to a positive impact of mineral supplements on the general health status in poultry. Minerals are important nutraceuticals required for the optimum health and physiological functions. The effectiveness of the use of microelements is an essential topic in modern poultry feeding. In addition, the advanced knowledge of the importance of microelements in the reproduction and immunological processes and the variable content of minerals in feed ingredients have led to their addition to poultry diets in commercial practice in high quantities with a large safety margin often exceeding the birds’ requirements (Saripinar-Aksu et al. 2012). Post-hatch, nutrients can have a prolonged effect on general health, broiler performance and tissue mineralization. Therefore, the use of nutraceuticals such as minerals is even more important where antibiotics are banned in diets completely.

In poultry, minerals are required as a part of an activator of hormones and enzymes, for the skeleton and eggshell formation and replacement, and for the maintenance of acid-base balance (sodium (Na), potassium (K) and chloride (Cl)) and osmotic homeostasis (Ravindran 2010). Recently, the use of minerals in an organic form (metal ion + amino acid ligand, chelated amino acids, proteinases) has increased. The application of organic mineral sources in poultry nutrition may prevent minerals from creating indigestible complexes with some dietary components and reciprocal mineral antagonisms in the intestine that could decrease their absorption rate (Świątkiewicz et al. 2014). Organic minerals in small amounts can be added to bird diets as minerals are better assimilated by poultry than mineral salts (Nollet et al. 2007; Ravindran 2010). A novel form of mineral supplementation to the poultry diet is also biomass enriched with microelements using the biosorption process. As it was shown in several reports, microelements such as Zn(II), Cu(II), Mn(II), Co(II) and Cr(III) were better bioavailable to laying hens from this feed additive than from inorganic salts (Michalak et al. 2011; Witkowska et al. 2014; Saeid et al. 2016). Researchers studied the antioxidant property of dietary supplementation of organic and inorganic Zn, Cu and Mn on white strain laying hens and results showed that both forms of zinc, copper and manganese helped in mitigating oxidative stress in laying hens (Bulbul et al. 2008).

Poultry requires macro elements as well as trace elements in the diet. Macro-minerals such as calcium (Ca) and phosphorus (P) are the most abundant elements in the body. This group also includes chloride (Cl), magnesium (Mg), potassium (K), sodium (Na) and sulphur (S). The required content of these elements in the diet is usually higher than 100 mg/kg feed (Ravindran 2010). Trace minerals such as manganese (Mn), selenium (Se), copper (Cu), iron (Fe) and zinc (Zn) are necessary for chicken development because these are active in many metabolic pathways – they are co-factors of enzymes and components of larger molecules (Ravindran 2010; Faria et al. 2020). They are required in poultry diet in trace amounts, usually about 0.01% (Ravindran 2010). Trace minerals participate in physiological functions necessary to sustain life, including growth, reproduction, immune system function, energy metabolism and bone formation (Bao et al. 2007; Dibner et al. 2007).

### 4.1. Calcium and phosphorus

Calcium (Ca) is a major element in poultry nutrition. It is an important component for mineralization of bones and shells, blood-clot formation and muscle contraction (Talpur et al. 2012). Bintvihok and Kositcharoenkul (2006) pointed out that dietary Ca addition had a positive effect on BWG. Driver et al. (2006) stated that the poultry diet containing 0.80% Ca enhanced quantity and quality of carcass of birds. Lack of calcium ions in bones led to the deterioration of the skeletal structure and reduced bone strength (Kwiatkowska et al. 2017). Coto et al. (2008) reported that the optimal ratio of available calcium and phosphorus in broiler feed is 2:1 that is conditioned by strong interactions between these elements. It should be underlined that phosphorus should be in the form of non-phytate which is biologically available to poultry. This element in the phytate-phosphorus form, commonly present in plant-derived ingredients is poorly utilized by poultry due to the lack of digestive enzyme – phytase (Ravindran 2010). There are studies conducted to assess the effect of high non-phytate phosphorus (NPP) diets, low NPP diets and administration of exogenous phytase on the growth performance, blood metabolites, phosphorus retention in plasma, activity of plasma alkaline phosphatase, bone characteristics and tibia P content in starter and broiler birds. Supplementation of phytase exogenously through the diet improved growth performance, bone parameters and noticeable P retention in growing broilers even when they were supplied with phosphorus poor diets (Baradaran et al. 2017). Also, phytase increased the availability of P for the animal to be used for biochemical functions in the body (Abd El-Hack et al. 2018a). Phosphorus is a necessary mineral for poultry and plays a significant role in the hard and soft tissues of the body (Underwood and Suttle 1999). Its requirements in poultry are affected by various factors, including the dietary level of calcium. The use of phosphorus is of growing concern with regard to the sustainability of broiler chickens production since high phosphorus excretion can cause eutrophication (Valable et al. 2018). Rao et al. (2006) pointed out that both phosphorus and calcium co-exist in many biological functions, but the dietary requirement of these minerals is interdependent. Calcium possesses the main role in the improvement of the skeletal structure, egg shell and blood cloth formation of poultry. Synoptically, there is a symphony in action between calcium and phosphorus in the body.

### 4.2. Trace minerals

Mn, Zn and Cu are structural constituents and catalytic of the antioxidant enzyme - superoxide dismutase (SOD) and also act on immunity mediators such as thymus peptides, cytokines and enzymes (Silva et al. 2015). Zn and Mn are co-factors involved in the carbonates and mucopolysaccharides synthesis which is necessary for bone formation (Świątkiewicz et al. 2014).

#### 4.2.1. Manganese

Mn contributes to the metabolism of carbohydrates, lipids, and amino acids (Crowley et al. 2000; Suttle 2010). It participates in many biochemical processes by activating enzymes such as glycosyl transferase, SOD and pyruvate carboxylase (Suttle 2010). It plays an important role in bone development, growth, optimal egg shell quality and perosis prevention (Lu et al. 2007; Olgun 2017). Also, it is a main component of the Mn-SOD that safeguards cells from oxidative stress (Li et al. 2011). In poultry, Mn is important for the eggshell formation and can clearly affect the egg shell quality. Some researchers have recommended the use of organic sources of Mn that substantially affected egg shell quality and performance (Yildiz et al. 2011; Sun et al. 2012). Olgun (2017) stated that the dietary addition of 90 mg Mn/kg feed prevented various disorders such as perosis, maintained normal development of broilers and improved egg shell quality parameters of laying hens. The supplementation of 12 mg Mn/kg feed from both sources (inorganic or organic) was sufficient to provide optimum broiler performance (Mwangi et al. 2019). Thus, Mn in poultry nutrition is crucial due to its role in egg shell and bone development and metabolism of nutrients.

#### 4.2.2. Zinc

Zn contributes in the maintenance of the immune function (Kidd et al. 1996), the growth performance (Liu et al. 2011) and the skeletal development of broiler chickens. Abd El-Hack et al. (2017b) stated that Zn possesses many roles as an antioxidant agent, and associates in the hormone function including pancreatic (glucagon and insulin), growth and sex hormones. Zhang et al. (2018) clarified that supplementing Zn in the starter and grower diets at levels of 40 and 32 mg/kg feed, respectively, promoted the growth performance of broiler chickens and reduced excretion of Zn in the environment. Zinc plays a useful role in the tissues of the pancreas that prevents oxidative damage and activates the secretions of the pancreas from digestive enzymes and thus stimulates the digestion of nutrients (Sahin et al. 2005). Zago and Oteiza (2001) indicated that Zn as a significant element of the antioxidant defense network inhibits membrane damage from oxidation and also it can partially stop the formation of free radicals and other reactive substances. Zinc participates directly in metabolic pathways and is a key component of cellular defense against oxidative stress as an integral part of cytosolic Cu/Zn SOD (Zago and Oteiza 2001; McDowell 2003). Saleh et al. (2018) exhibited that dietary organic zinc supplements improved growth performance, humoral immunity, antioxidant properties, nutrient digestion and zinc content in raw meat, and reduced lipid peroxidation in broiler meat. The addition of organic zinc had a positive influence on the immunological capacity by improving immunoglobulin (IgA, IgM, and IgG) levels and may also improve cellular response of broilers (Moghaddam and Jahanian 2009; Feng et al. 2010). Dietary supplementation of zinc-methionine (Zn-Met) at 25, 50, 75 or 100 mg/kg diet increased Zn status and reduced blood triglyceride, LDL-cholesterol and resulted in improving the antioxidant capacity of laying hens (Abd El-Hack et al. 2018b). Finally, zinc plays multiple roles in metabolism, immune response and antioxidant systems of poultry.

#### 4.2.3. Copper

Cu is involved in both humoral (facilitates antibody production) and cell-mediated immunity (assists in eliminating invading bacteria). It shows immunostimulating action and supports maintaining appropriate microbiological balance in the digestive tract (Makarski et al. 2014). Thus, it has been used in poultry production as a nutritional supplement due to its microbiological activities and the ability to increase BW (Wang et al. 2008). Copper as a feed additive has a helpful effect on the BWG, FCR and modification of the bacterial microflora in the gut (Ruiz et al. 2000). Supplementation of copper sulfate (up to 200 mg/kg feed) to broiler diets had a beneficial influence on growth performance (Hashish et al. 2010). Xia et al. (2004) indicated that 150 mg copper sulfate/kg feed of broiler chicks had an affirmative impact on BWG that may be the result of the significant decline in the total pathogenic organism in the gut. Samanta et al. (2011) described that supplementation of copper in broiler chickens’ diet improved growth performance, as well as reduced plasma triglyceride and cholesterol, and meat cholesterol. Kumar et al. (2013) stated that the dietary addition of copper is useful for performance and blood biochemical parameters of broiler chicken. Recently, Yang et al. (2018) indicated that the dietary supplementation of Cu at levels of 8.77 and 11.6 mg/kg feed can improve growth and carcass yield in growing goslings from 28 to 70 days of age. Moreover, copper is a pro-oxidant in its unbound form (Diplock et al. 1998). Also, copper is a constituent of SOD and defends living organisms against reactive oxygen species. Copper salt at a pharmacological dose decreased cholesterol 7α-hydroxylase activity (Konjufca et al. 1997; Yang et al. 2018). Copper is involved in iron transport and metabolism, and the formation of red blood cells. In this regard, Samanta et al. (2011) confirmed that supplementation of copper is an effective way to improve haematological parameters in broiler chicken. Thus, copper is a micronutrient involved in many physiological processes and immunity, and it is necessary for optimal health and growth of poultry.

### 4.3. Iron

Iron (Fe) helps in transportation and storage of oxygen and enhances protein metabolism, energy supply and processes of anti‐oxidation and immunization inside the body (Drygalski and Adamson 2013; Abbaspour et al. 2014). Iron is used in the feed industry as a feed supplement in poultry diet (Xie et al. 2019a). It is important as a co-factor for the function of many enzymes (Lozoff et al. 2006) and is an essential structural co-factor for numerous proteins (Scott et al. 2008). Bess et al. (2012) illustrated that supplying a suitable level of Fe (60 mg/kg feed) in broiler breeder diet can improve the productive performance. Some studies have suggested that using organic iron supplements to poultry feed could improve their immunity and antioxidant capacity (Xie et al. 2019a, 2019b). Also, Shinde et al. (2011) stated that organic sources of iron supplementation improved the performance of broilers. Nikonov et al. (2011) described that iron supplementation to broiler breeders hens diets improved their performance. The *in ovo* injection of Fe can improve BW and BWG and reduce serum cholesterol and total lipids of chickens and can also produce more healthy food for human consumption (Mogahid et al. 2019). In summary, iron helps in the transportation and storage of oxygen, participate in energy supply, metabolization of protein, and improves the immunity and antioxidant capacity.

### 4.4. Selenium

Selenium (Se) is an important trace nutrient for the maintenance of growth and health of humans and animals (Kieliszek and Błażejak 2016). When supplemented to the diet, it maintains the high reproductive and productive performance of poultry (Papazyan et al. 2006). The rate of hatchability and fertility in chicken were improved by organic selenium supplementation (Rizk et al. 2017). Also, selenium boosts bursa and thymus weight and increases immunity (Hussain et al. 2004). Supplementation of organic selenium in broiler diets improved FCR and reduced drip-loss, leading to enhancing the economic gain and meat quality (Deniz et al. 2005). Organic selenium in the poultry diet is also associated with the increased hatchability and fertility in breeders, lower mortality and better FCR in broilers (Surai 2006). Selenium has an important role in promoting health because it is one of the most active natural antioxidants. Supplementation of dietary selenium enhanced catalase, SOD and GSH-Px activities and lessened oxidative stress and lipid peroxidation biomarkers in broilers (Cai et al. 2012). The application of organic selenium may be advantageous in improving certain variables of performance and meat quality (Ravindran and Elliott 2017). In addition, selenium maintains tissue integrity, protects the body from oxidative stress and prevents the occurrence of diseases that have oxidative stress as a triggering factor (Pappas et al. 2008). Also, selenium-dependent glutathione peroxidase (Se-GSH-Px) enzyme is an important factor in the antioxidant system of the semen especially under stress conditions (Ebeid 2009). Selenium is crucial for the regulation of gene expression e.g., GSH-Px gene that is involved in the antioxidant system, immune regulation and biological functions (Habibian et al. 2014). Selenium may not be directly involved in inhibiting ROS formation, but indirectly through enzyme actions in which it serves as a co-factor (Horvath and Babinsky 2018). Experiments showed that incorporation of the organic and inorganic form of Zn and Se through the addition of rosemary, hydroxytyrosol, pomegranate, grape and harpagophytum extracts in broiler diet enhanced the nutritional quality as well as the shelf life of chicken nuggets. Moreover, if phenolic compounds were added together with Zn and Se, they helped in maintaining the sensory quality by lowering the growth of microorganisms and diminishing the protein and lipid oxidation (Martínez et al. 2020). Thus, Se revealed strong nutritional and biological effects on improving the productive and physiological performance of poultry.

### 4.5. Iodine

Iodine (I) is a trace element with several biological functions. One of the most important is the proper functioning of the thyroid gland as iodine is a constituent of its hormones (triiodothyronine and thyronine) that play an essential role in regulation of metabolism, cellular oxidation and intermediary cell activity (Van Middlesworth 1996; Delange 1998; Lewis 2004). Furthermore, there are some functions that depend on the supply of iodine for any organism such as circulation and muscular systems, maturity processes of cells and tissues, reproduction properties, functions of the nervous system, and secondary skin product formation (Travnicek et al. 1997; Delange 1998; Liu et al. 2001). The enrichment of products of animal origin with iodine can be achieved through supplying many dietary iodine sources (NaI, KI, and Ca (IO_3_)_2_) and iodine levels in the animal diet (Słupczyńska et al. 2014). In the poultry diet, iodine is supplemented mainly within the mineral premix in the form of Ca (IO_3_)_2_, KI, or iodized salt. Iodine is a very essential microelement in laying hens’ feeding, and has a strong impact on growth performance of birds (Opaliński et al. 2012). The addition of iodine (2 mg/kg feed) to the drinking water significantly boosted broiler growth (Stanley et al. 1989). The effectiveness of iodine in improving the productive performance is due to its major role in regulation of metabolism.

### 4.6. Chromium

Chromium (Cr) plays an important role in poultry health and nutrition, as well as augments growth performance. It is a potent antioxidant and hypocholesteremic agent. Chromium is known to decrease cholesterol, increase high-density lipoprotein cholesterol and improve nutrient digestion (Haq et al. 2016). Dietary chromium has valuable impacts on immune response and antioxidant defense system (Farag et al. 2017). Chromium also improves FCR, influences weight gain, increases relative organ weight and muscle development (Haq et al. 2016). There are beneficial chromium impacts on the reproductive and productive performance, as well as physiological traits (Sahin et al. 2005). Chromium can also alleviate the effect of stressors such as environmental, nutritional, physiological, physical stress, etc. in poultry production (Chandrasekar and Balakrishnan 2019). Arif et al. (2019) clarified that better performance and weight gain may be achieved when chromium propionate is added to the broiler diet at the rate of 400 ppb. Dietary chromium supplementation boosted immune functions of chickens vaccinated with Avian Influenza Virus (AIV) (Lu et al. 2019). Deficiency of this element disrupts carbohydrate and protein metabolism (Haq et al. 2016). In conclusion, chromium is necessary for improving productive performance in poultry due to its important functions in growth, metabolism and reduction of lipid and protein peroxidation.

A summary of the implications and beneficial effects of different amino acids, vitamins and minerals as nutraceuticals in poultry is presented in Table 1.

**Table 1.** Summary of the implications and beneficial effects of different amino acids, vitamins and minerals as nutraceuticals in poultry.

Nutraceutical type and dose	Poultry species	Implication/Conclusion	References
**Amino acids**			
Trp, Ile, His, Val, Leu, Arg, Gly and Phe	Male broilers	Adding the essential amino acid mixtures to the low CP diets improved the performance but did not completely overcome the adverse effects of the low CP diets	Waldroup et al. 2005
Threonine (0.4, 0.5, 0.6, 0.7, 0.8, 0.9, 1.0 and 1.1%)	Ross 308 males	Gut functionality like microvilli height, epithelia thickness and crypt depth was improved with even higher levels of dietary standardized ileal digestible threonine level	Zaefarian et al. 2008
Arginine (2% L-arginine)	Broilers	Arginine increases specific immune response against Infectious Bursal Disease	Tayade et al. 2006
Threonine, valine and tryptophan	Laying Japanese quails	Reducing the CP level in a diet supplemented with crystalline amino acids is a valuable strategy for decreasing feeding cost and mitigating ammonia emission	Alagawany et al. 2014
Threonine and methionine	Broilers	Performance and immune system were improved at higher dietary threonine and methionine levels	Yaqoob and Ali 2018
Threonine (0.0 (control group), 0.25, 0.50, 0.75 and 1.00 g/kg diet)	Broilers	Adding threonine in the diet may promote the growth of immune organs, encourage the antibodies synthesis and mitigate the immune stress caused by Newcastle disease virus or *E. coli* challenge	Azzam El-Gogary 2015
Arginine (0%, 0.45%, 0.90%, 1.35%, and 1.80% Arg)	Broiler	The addition of arginine in the diet could improve the growth performance of broiler chickens at 42 days of age	Xu et al. 2018
Lysine and methionine	Male broiler	There are positive effects on meat yield and growth performance in response to supplemental amino acids in diets from 21 to 41 days of age	Zhai et al. 2016
L-Methionine (8 g/kg diet)	Rabbits	Reduced detrimental impacts of aflatoxinB1 on growth, immune and antioxidant status	Reda et al. 2020
Three levels of Met + Cys (74%, 77% and 80%) of digestible lysine	Broiler chickens	DL-Met and L-Met are equally effective as a source of methionine for broilers	Rehman et al. 2019
Threonine (0, 300, 600 and 900 mg/ kg diet)	Broiler chickens	A significant improvement was observed in performance indices of birds fed diet enriched with threonine compared with the control	Al-Hayani 2017
Threonine, arginine, and glutamine	Broiler chickens	May help to minimize over-activation of the innate immune system, which is the most expensive in terms of energy and nutrients, as well as improve the intestinal microbiota	Bortoluzzi et al. 2018
Apparent and standardized ileal amino acid digestibility	Broiler chickens	Increasing dietary levels of highly digestible amino acids may help compensate for malabsorption through the stages of intestinal challenge	Adedokun et al. 2016; Rochell et al. 2016
Methionine (a control (0.49% methionine) or a deficient (0.28%)	Cobb500 broiler male parent	A methionine deficiency affects essential amino acids digestibility and cysteine, but not the methionine digestibility. The alterations in digestibility are reflected in the expression of mRNA of amino acid transporters across different tissues	Fagundes et al. 2020
Threonine (100, 110, and 120% of NRC recommendation)	Mixed sex broilers (Ross-308)	Use of threonine, above NRC requirements, resulted in a better growth rate, feed utilization and carcass quality, gut health, increased ileal digestibility of amino acids and protein, and immunity	Ahmed et al. 2020
Threonine (i.e., 100%, 110% and 120% of Ross recommendations)	Broiler chickens	An improvement in feed intake through the grower period and an improvement in body weight (BW)throughout the grower and overall period, whereas a better feed conversion ratio through the starter period in birds fed 10% extra threonine in comparison with the control diet	Zarrin-Kavyani et al.^2018^
Threonine (100% NRC specification, 100, 110, 120 and 130% threonine of Vencobb-400 strain specification)	Broiler chickens (Vencobb-400)	The immune organs weight was improved with threonine supplementation	Debnath et al. 2019
3.0 g threonine/kg feed	male chicks	The level of intestinal cytokines in lipopolysaccharide-challenged chickens was reduced by threonine addition	Chen et al. 2018b
**Vitamins**			
Vitamin E	Broiler chickens	improvement of the immune response and antioxidants concentration in the liver	Karadas et al. 2016
2 g α-tocopherol acetate/kg feed	Broilers	Increase in carcass mass and decrease in the abdominal fat of broilers	Zaboli et al. 2013
Vitamin E	Broiler chickens	A significant influence on the chicken meat quality by reducing juice drip and increasing WHC of meat	Zdanowska-Sasiadek et al. 2016
Vitamin A (16,000 IU/kg feed)	Hy-sex	Improvement of productivity performance parameters	Abd El-Hack et al. 2017a
Vitamin C (200 mg/kg feed)	Broiler chickens	Protection against the risk of high density by improved final BW, reduction of mortality and downregulation of HSP70expression level in the liver	Shewita et al. 2019
Vitamin C (200 mg/kg feed)	Commercial broilers	Improvement of the immunity of broilers	Lohakare et al. 2005
Vitamin A (0, 8,000 and16,000 IU/kg diet) and vitamin E (0, 250 and 500 mg/kg diet)	Bovans Brown laying hens	Both vitamins play a role in alleviating the harmful impacts of high ambient temperature. Use of 16,000 IU vitamin A with 500 mg vitamin E /kg diet is preferable for obtaining better production of birds exposed to heat stress	Abd El-Hack et al. 2019
Vitamin E (0, 250 mg/kg diet)	Growing Japanese quail	Useful in partly alleviating the adverse impacts of cadmium	Abou-Kassem et al. 2016
VitaminE	Laying hens	Prevents unsaturated lipid oxidation within cells, therefore protecting the cell membrane from oxidative damage induced by ROS	Mahrose et al.^2012^
Vitamin E (200 mg/kg feed)	Male chickens	Enhanced semen quality traits, including the spermatozoa count and motility, and reduced the dead spermatozoa, under heat stress conditions	Ebeid 2012
Vitamin E (100 mg/kg feed)	Poultry ganders	Improved ejaculate volumes, percentages of viable sperm and sperm concentrations and lowered percentages of spermatids	Jerysz and Lukaszewicz 2013
Control with additional 3,000 or 9,000 IU25-hydroxyvitamin D_3_/kg feed, 3,000 or 9,000 IU vitamin D_3_/kg feed, 3,000 or 9,000 IU vitamin D_2_/kg feed	Lohmann white laying hens	Irrespective of forms, the apparent total tract digestibility of calcium was higher in diets enriched with vitamin D. The apparent total tract digestibility of phosphorus was higher in 3,000 IU/kg of vitamin D_2_ compared to the other treatments. The utilization of calcium and phosphorus by laying birds can be enhanced by the addition of different sources of vitamin D in rations	Adhikari et al. 2020
Ca (3.0, 3.5, 4.0, and 4.5%) and 25OHD3 (0, 69, and 138 μg/kg feed)	Lohmann LSL-lite layers	Use high levels of calcium and 25OHD3 improved bone strength and decreased risks related to morbidity, leg weakness and mortalities	Kakhki et al. 2019
**Minerals**			
Copper sulfate (200 mg/kg feed)	Broiler	Useful influence on the growth rate	Hashish et al. 2010
150 mg copper sulfate/kg feed	Broiler chicks	Improved live BW gain that may be the result of the significant decline in the total pathogenic bacteria the gut	Xia et al. 2004
Copper(8.77 and 11.6 mg/kg feed)	Goslings	Improved growth and carcass yield from 28 to 70 days of age	Yang et al. 2018
12 mg Mn (inorganic or organic)/kg feed	Broiler	It was sufficient to provide optimum broiler performance	Mwangi et al. 2019
Zn-Met (25, 50, 75 or 100 mg Zn-Met/kg diet)	Hisex Brown laying hens	Increased Zn status and reduced blood triglyceride, LDL-cholesterol and resulted in improving antioxidant capacity	Abd El-Hack et al. 2018b
Chromium propionate with inclusion levels of 0, 200, 400, 800 and 1600 ppb.	Male ROSS-308 broilers	Better performance and weight gain may be achieved if chromium is added in broiler diets at the rate of 400 ppb	Arif et al. 2019
Selenium (0, 0.25, 0.50 mg/kg feed)	Bovans laying hens	Hemoglobin and lymphocytes were increased with increasing dietary Se level in layer reared under heat stress conditions	Abd El-Hack et al. 2017a
Selenium	Poultry males	Plays an important role in semen quality and is related to the high proportion of polyunsaturated fatty acids in avian semen and its susceptibility to lipid peroxidation	Surai et al. 1998a
Organic Se	Cockerel	Dietary supplementation of organic Se in the cockerel’s diet increased (more than double) Se concentration in the semen; have a beneficial effect on the antioxidant defense in various tissues including sperm	Surai et al. 1998a,b
Selenium ( 0. 3 mg Se/kg feed)	Male chickens	Use of Se in the diet of male chickens increased the activity of GSH-Px in the liver, testes, spermatozoa and seminal plasma	Surai etal. 1998c
Organic Se (0.3 mg/kg feed)	Male chickens	Under high ambient temperature (33-36 °C in poultry farm), use of organic Se in the cockerel diets improved the GSH-Px activity and semen quality (motility and sperm count) and reduced the dead sperms count in a dose-dependent manner	Ebeid 2009
Organic Se (0.3 mg/kg feed)	Male chickens	Enhanced semen quality traits, including the spermatozoa count and motility, and reduced the dead spermatozoa, under heat stress conditions	Ebeid 2012
Selenium (0.3 mg/kg feed)	Poultry ganders	Improved ejaculate volumes, percentages of viable sperm and sperm concentrations and lowered percentages of spermatids	Jerysz and Lukaszewicz 2013
0, 0.5, 1.0 or 2.0 mg Se (sodium selenite)/kg diet	Hy-Line roosters	The highest activity of GSH-Px and lowest content of MDA in blood and testis was recorded in the treatment of 0.5 mg/kg	Shi et al. 2014
Dietary Se deficiency (0.033 mg of Se/kg feed) in comparison with the control	Hy-line cockerels	Exerts harmful impacts on reproductive organs and the extrinsic and intrinsic pathways and the upstream regulators, like Bcl-2 and p53 are all involved in Se deficiency-induced testicular apoptosis	Huang et al. 2016
0.15 mg Se/kg feed from sodium selenite, Se-enriched yeast (Se-yeast) or SeMet	Broiler breeders	Apart from sodium selenite, Se-yeast or SeMet increased the activity of thioredoxinreductase-1 in the kidney and liver of breeders and their offspring, but not the activity of GSH-Px1	Yuan et al. 2012
Se 0.13 mg/kg feed with 0.4 mg Se in the form of sodium selenite (SS) or Se-yeast/kg feed for 9 months	Hy-Line Brown	Increased Se content of the e.g., g from 5.1 µg in the basal diet group to 14.4 and 22.7 µg, in SS or Se-yeast, respectively	Cobanová et al. 2011
Organic selenium (0.5 mg/kg diet)	Poultry breeders	A reduction in mortality with selenium supplementation; increase in e.g., g production, hatchability, and percentage of settable e.g., gs	Rajashree et al. 2014
Organic selenium (2 vitamin E levels (30 and 120 mg/kg feed) and two selenium sources (sodium selenite and zinc-L-selenomethionine).	Broiler breeder	Promoted heavier hatchling weight until e.g., g production peak (33 weeks), but did not influence hatchling quality	Urso et al. 2015
25 or 75 mg ZnO/kg diet	Laying hens (Hisex Brown)	Dietary zinc addition up to 75 mg/kg used as an effective supplement to improve antioxidant ability and zinc status in laying hens	Abd El-Hack et al. 2020b

## 5. Advances in delivery of nutraceuticals

Various advanced delivery options are needed to be explored for efficient utilization of amino acids, minerals and vitamins in poultry feeding (Abd El-Hack et al. 2017c; Alagawany et al. 2018c; Saeed et al. 2019). These delivery systems enable efficient supply of nutraceuticals, their increased bioavailability, reduce the incompatibility, protect against degradation of essential constituents, minimize doses and side effects, besides facilitating prolonged beneficial effects of nutraceuticals (McClements 2012; Aklakur et al. 2016; Helal et al. 2019; Jampilek et al. 2019). Various delivery forms are being evaluated for better results. Chelated forms of nutraceuticals, organic and inorganic types (Khatun et al. 2019), nanoformulations (Aklakur et al. 2016; Gangadoo et al. 2016), micronized particles (Tufarelli and Laudadio 2015) and encapsulated nutraceuticals are some of the delivery forms of few nutraceuticals that have shown promising results or are under evaluation or have bright future prospects (Aklakur et al. 2016; Helal et al. 2019; Jampilek et al. 2019). However, each of the component or subcomponent of nutraceuticals may vary in delivery forms or the effect of delivery system on the health and production performance of poultry birds can be different hence each of them requires respective evaluation.

Amino acids are being used in different forms to achieve better results in terms of production performance. Nowadays, individual amino acids in pure forms are commercially available (Ravindran 2010). This also minimizes the loss through excretion via waste products. Due to the lower absorption of conventional forms in the gut, higher doses of amino acids in poultry feed are required, which increases the cost of farming. Methionine has been evaluated as methionine hydroxyl analogue, DL-methionine and L-methionine. No significant differences were found in these forms on health, performance, and the quality composition of breast meat of birds. L-methionine caused the highest protein and the lowest fat content in meat (Ullirich et al. 2019). Altering levels of amino acids in the diet may affect performance and health status in poultry. L-carnitine and excess lysine and methionine in the diet did not affect feed intake in starter birds but in finisher birds it did. L-carnitine supplementation had no effect on IgM titer but had an effect on serum antibody titers in broilers vaccinated against Newcastle disease and Gumboro's disease (Ghoreyshi et al. 2019).

Minerals in typical poultry feed formulations are delivered mainly in the inorganic form, for example calcium as limestone, shell grit; calcium and phosphorus as dicalcium phosphate, defluorinated rock phosphate, bone meal; sodium as salt, sodium bicarbonate and trace minerals in the form of premixes (Ravindran 2010). But novel forms are also introduced. Minerals are being used in chelated and organic forms for better delivery, absorption, utilization and conversion efficiency. Methionine chelate or yeast proteinate-based supplement of copper, iron, manganese and zinc have been utilized and their effect on broiler growth performance, distribution in the tibia and excretion into the environment have been evaluated (Singh et al. 2015). The inorganic and organic forms of minerals affected growth performance, carcass characteristics, bone quality, and chemical composition of poultry birds. Chelated forms of minerals are also being used for better results (Zhao et al. 2016). Increased levels of minerals like zinc along with phytase in diets of poultry lead to better utilization of nutrients and enhance production performances (Akter et al. 2017). Organic forms of minerals have numerous advantages over the inorganic forms, for example, proteinate and propionate organic trace minerals were found to be more effective in improving performance, immune response, and profitability in broiler birds than inorganic trace minerals (Khatun et al. 2019). They may affect bioavailability and hence tissue levels thereby determining the actual concentration at sites of requirement or action. Biomass enriched with minerals by biosorption has similar properties to chelates. The bioavailability of microelements from this form (enriched macroalgae, microalgae, soybean meal) to laying hens was higher than in the case of traditionally used inorganic salts. Moreover, this type of biological feed additives enriched eggs with microelements and additionally influenced positively egg shell thickness, egg weight, as well as BW of hens (Michalak et al. 2011; Witkowska et al. 2014; Saeid et al. 2016).

A novel approach in poultry production is the application of nanoparticles, which can serve as a platform to incorporate nutrients into the body. They enable direct transportation of active compounds to target organs, avoiding their fast degradability and encourage several health benefits. Nanoparticles in poultry production include elements such as silver, selenium, copper, and gold (Gangadoo et al. 2016). For example, nanoselenium has been found to be more effective in improving production performance, meat quality and minimizing oxidative stress in broiler chicken when compared to selenomethionine and inorganic Se. This can result from the efficient retention of this novel delivery form of selenium within body, better utilization, enhanced conversion efficiency and improved antioxidant defense (Ibrahim et al. 2019). Similarly, nanoparticles of gold, silver and copper have shown potential roles in improving production performance, antioxidant defense system, and immunological response against diseases. Additionally, increased BW, improved feed intake and conversion efficiency, protein synthesis, and modulation of microbiota in poultry birds was observed (Anwar et al. 2019). Finally, the improvement in poultry growth and performance by adding amino acids or mineral components in such novel form is attributed to their higher bioavailability.

The dietary supplementation of cyanocobalamin showed no effect on feed intake, daily weight gain and feed conversion efficiency, but it improved hematological parameters including red and white blood cells and platelet counts (Ahmad et al. 2019). Some vitamins based formulations have a preventive and curative effect on infections in poultry. One such similar formulation of vitamin C has shown prophylactic and therapeutic effects on *Salmonella* Enteritidis and necrotic enteritis infections in broiler chickens (Hernandez-Patlan et al. 2019). Nanoformulations of vitamins A, D, E have shown stability, better delivery, improved serum levels, sufficient bioavailability, and enhanced *in vivo* and *in vitro* performance (Jampilek et al. 2019) thus having promising properties as nutraceutical delivery systems for poultry as well.

## 6. Designer and functional foods concepts

Nutraceuticals could serve as functional food by providing various beneficial health impacts (Martirosyan and Singh 2015). Amino acids, minerals and vitamins as natural components in food or other ingestible forms provide benefits to the human body such as preventing or curing diseases and improving physiological processes and immunity. Various concepts are developed to utilize these nutraceuticals in poultry production and health, including developing designer eggs, designer meat, designer grains, designer proteins and designer food enriched with micro- and macro-nutrients. To develop such functional food, biofortification and nutrification technology is employed (Rajasekaran and Kalaivani 2013; Dhama et al. 2014b; Laudadio et al. 2015; Alagawany et al. 2018c). Poultry meat and eggs can be considered as functional food because beneficial nutrients from the feed are transferred into poultry products (Perić et al. 2011). Michalak et al. (2011), Saeid et al. (2016) and Witkowska et al. (2014) showed that using algae biomass (both seaweeds and macroalgae) or soybean meal enriched with microelement ions via biosorption it was possible to obtain eggs biofortified with these elements.

Stephen DeFelice used the word nutraceuticals in 1989 for nutritional substances which can be consumed as pharmaceuticals too. Hasler (2002) also stated that nutraceuticals besides being a nutritional component must contribute to some therapeutic or disease preventive effect. Vitamin D, vitamin E, monounsaturated fatty acids (MUFA) as oleic acid present in olive oil; polyunsaturated fatty acids (as alpha-linolenic acid) found in walnuts; eicosapentenoic acid (EPA) and docosahexaenoic acid (DHA) mainly present in oily fish; selenium, green and black tea, cocoa, coffee, cinnamon, fenugreek, guava, lycopene, soybean, garlic, various flavonoids and antioxidants usually present in fruits and vegetables, are some relevant examples of nutraceuticals exerting chief impact on health, especially gastro-intestinal health (Cencic and Chingwaru 2010; Serafini and Peluso 2016; Alkhatib et al. 2017).

It is documented that functional food containing acetylcholinesterase inhibitors along with potential antioxidants can be a treatment option against neurological disorders like Alzheimer’s disease and/or dementia. Phenolic compounds like 2-hydroxy-4-methoxybenzaldehyde and 4-hydroxy-3-methoxybenzaldehyde present in *Hemidesmus indicus* and *Vanilla planifolia* due to their acetylcholinesterase inhibitory activity are suitable candidates for functional food production, which can be used in the treatment of neurological disorders (Kundu and Mitra 2013; Matirosyan & Singh 2015; Wilson et al. 2017). Researchers have studied the effect of feeding bioactive peptides on the antioxidant potential of broiler breast meat. The findings suggested that dietary supplementation of bioactive peptides obtained from fish waste helped in increasing the antioxidant potential and shelf stability of broiler meat when they were stored even under frozen conditions. This study also concluded that natural bioactive peptides in the form of dietary nutraceuticals can be a suitable substitute to the commercial chemical anti-oxidants used in poultry feeding to keep the quality of meat and by-products such as nuggets (Aslam et al. 2020). In another experiment, researchers used diets supplemented with linseed oil, echium oil, fish oil and/or algal biomass to study their health promoting effects on various parameters of immune response including total lipid profile, lymphocyte proliferative effects and NK cell activity among thymocytes and splenocytes in one day old male broiler chickens. Being enriched source of DHA and n-3 polyunsaturated fatty acids, these diet components improved the NK cell activity in splenocytes, enhanced fatty acid production in all tissues and also augmented the lymphocyte proliferation (Al-Khalaifah et al. 2020).

Nutraceuticals can be employed to develop designer and functional food. Manipulation of nutraceuticals in the poultry diet can help in altering the composition of poultry products that can be adjusted to the demands and health requirements of consumers. Fatty acid or cholesterol, mineral, vitamin, and/or amino acid composition of eggs or meat can be modified by dietary nutraceuticals that will serve as a basis for producing designer and functional products, having beneficial health applications in addition to nutritional applications (Rajasekaran and Kalaivani 2013; Alagawany et al. 2018b). Fatty acid composition of meat or eggs can be manipulated by the addition or deletion of omega fatty acids (omega-3 (n-3) and omega-6 (n-6) fatty acids) in poultry diet. This maintains a proper balance of these fatty acids and thus can help to avoid serious complications associated with the imbalance of the low density and high density lipopolysaccharides like diabetes mellitus, hypertension, increased bleeding risk, obesity, and coronary artery diseases (Fernandez 2015; Ibrahim et al. 2018; Alagawany et al. 2019). In the work of Michalak et al. (2020), it was shown that the application of an extract, obtained from microalga *Spirulina platensis* by supercritical fluid extraction, generated biofortified eggs with polyunsaturated fatty acids, including n-3 and n-6.

Low cholesterol diets are preferred nowadays for numerous beneficial health effects. They may also modulate immune and inflammatory responses that can be helpful in various diseases (Ibrahim et al. 2018; DiNicolantonio & O’Keefe 2018). Similarly, modulating sodium and potassium levels can help in the prevention and treatment of cardiovascular diseases like hypertension (Jahandideh et al. 2014; Fernandez 2015). This designer food can overcome the limitations and adverse effects associated with pharmaceuticals or chemical drugs along with being natural components of diets having nutritive qualities (Rajasekaran and Kalaivani 2013; Alagawany et al. 2018c). However, developing such a food, manipulation or modification of composition, and application in the diet or as supplements should be properly regulated to prevent any physio-biological adversary.

## 7. Conclusion and future prospects

Nutraceuticals are proved to be beneficial in poultry production both for supporting nutritional requirements and having pharmacological benefits, thus providing healthy and disease free physiological state. Being natural components of poultry diet, nutraceuticals can overcome side effects and limitations of chemical drugs. The demand for using an alternative natural feed additive such as nutraceuticals is essential especially after preventing using of antibiotics in poultry feed as growth factors. Currently, nutraceuticals are applied for boosting poultry growth rate, preventing diseases, and modulating immunity. Conversely, applying nutraceuticals for disease treatments has yet to be verified. Common nutraceuticals including amino acids, minerals and vitamins are vital for life processes of poultry. Having a role in numerous physio-biochemical mechanisms these nutraceuticals are involved in various essential growth and developmental processes. Modification of mineral, vitamin and amino acid composition of eggs or meat can help in developing designer or functional foods that can have potential applications in disease prevention and treatment. Nutraceuticals can be used for covering the gap between increasing the world’s population and production of high quality and low-cost animal feed. However, further large-scale studies are required in order to use nutraceuticals in commercial poultry production.

## Animal welfare statement

No ethical approval was required as this is a review article with no original research data.
